# The law of blast stress wave propagation and fracture development in soft and hard composite rock

**DOI:** 10.1038/s41598-022-22109-z

**Published:** 2022-10-12

**Authors:** Xiaohua Ding, Yuqing Yang, Wei Zhou, Wen An, Jinyu Li, Manda Ebelia

**Affiliations:** 1 School of Mines, China University of Mining and Technology, Xuzhou, 221116 Jiangsu China; 2 School of Mines and Mineral Sciences, Copperbelt University, 21692 Kitwe, Zambia

**Keywords:** Engineering, Physics

## Abstract

The process of blasting stress wave propagation and crack propagation is directly affected by the physical properties of the rock mass and internal joints in the rock. In soft and hard rock layers, the blasting process is more complicated since the blasting stress wave needs to penetrate two kinds of rocks with different physical properties and the interface between soft rock and hard rock. In this study, the modal transformation of stress waves at the interface of layered composite rock was analyzed, and the process was reproduced by finite element analysis. Furthermore, the development law of cracks was explored. The research results demonstrated that in the single blasting-hole model, a triangular crack area caused by reflected stress waves appeared at the rock interface of rock medium I near the blast hole. In rock medium II, the tensile crack generated by the interface wave appeared on the side away from the blast hole. Besides, the development of the tensile crack was associated with the incident mode of the blast stress wave and the incident angle. In the deep hole blasting model, the incidence of the detonation wave front from hard rock to soft rock promoted the fragmentation of the hard rock.

## Introduction

In the geological structure of the earth, most of the sedimentary rocks have a layered structure, and some metamorphic rocks also have a layered structure. The mechanical obstacles of layered composite rock mass should be tackled in the design and construction of engineering such as mines, tunnels, slopes, water conservancy and hydropower construction, and transportation^1–3^.

The composite rock mass is a naturally layered material composed of various properties, thicknesses, and components in different combinations in a certain order. The mechanical properties of layered composite rocks are significantly different from those of single homogeneous rocks^4^. Owing to the addition of soft rock, the loading conditions in the layered rock are changed. Stress attenuation occurs inside the rock, the attenuation strength mainly depends on the strength of the soft rock, and the initiation and propagation of cracks in the hard rock are inhibited^5,6^.

In the field of engineering blasting, the blasting effect is directly influenced by the inhomogeneity of the interlaced composite rock mass of soft rock and hard rock^7,8^. In soft and hard rock formations, the blasting process is more complicated because blasting stress waves need to penetrate two kinds of rocks with different physical properties and natural joint surfaces in the rocks. Regarding the explosion process of layered rock mass, Wang et al.^9^ investigated the effective velocity of reflected wave incidents on different rock masses. They revealed that the effective velocity of the reflected wave is determined by the incident frequency, joint stiffness, and impedance ratio. Daehnke and Rossmanith^10^ proposed the reflection and refraction stress coefficients of stress waves at different rock interfaces through theoretical studies. Additionally, the joint in the rock mass directly impacts the development of blasting cracks. Xu et al.^11^ and Chen et al.^12^ performed experiments to compare the effects of transverse and longitudinal joints perpendicular to the blast hole direction on crack development. The results suggested that the stress is concentrated at the end of the transverse prefabricated crack far from the explosion source, and explosion cracks develop along transverse cracks. In longitudinal prefabricated joints, stress waves reflect on the joint surface and create lamination cracks. Zhu et al.^13^ researched the effect of vertical and horizontal void joints on rock explosion cracks through numerical simulation. They unveiled that cracks always develop perpendicular to the joint surface. Although these experimental studies have drawn some conclusions on the influence of heterogeneous rock mass and joints on the blasting process, the propagation mode of stress waves and the development law of fractures in layered composite rocks remain unclear.

At present, there are three main theories about the development of blast cracks in rock mass: the theory of shock wave tensile failure, the theory of explosion pressure failure of explosive gas, and the theory of combined action of shock wave and explosive gas. The theory of combined action of shock wave and explosive gas is more accepted by scholars^14,15^. The shock wave first acts on the rock mass, causing initial fractures around the borehole; then, the detonating gas rapidly wedges into the initial crack, further promoting explosion-induced crack growth. Tang et al.^16^ observed the destruction process of the rock by the explosive gas using a high-speed camera. With the incompressibility of water, Xu et al.^17^ separated the effects of shock waves and explosive gases by detonating explosives in water. The test results suggested that the energy released by the detonating gas accounted for about 40–50% of the energy released by the explosive. Therefore, the effect of high-energy gas produced by blasting cannot be ignored on the development of cracks.

Numerical simulation is a crucial tool to study the blasting process. Various computational methods have been developed to simulate blast-induced cracks, including mesh-based methods (finite element method (FEM)^18^, extended finite element method (XFEM)^19^, discrete element method (DEM)^20^, smoothed particle hydrodynamics (SPH)^21^ and coupled methods (FEM-DEM)^22^ and DEM-SPH^23^. LS-DYNA is a general finite element code for calculating the dynamic response of structures to large deformations. LS-DYNA can handle the nonlinear dynamic response of structures through its explicit integration scheme and is particularly useful in simulating the failure process of materials under shock and explosion loads. This has been verified by many other researchers^24,25^. Through the experimental device of the Split Hopkinson Pressure Bar (SHPB), Wang et al.^26–28^ analyzed two main factors influencing the fracture of rock materials: maximum tensile stress and fracture energy. Wang et al.^29^ analyzed the rationality of these two factors in finite element analysis in LS-DYNA. Tao et al.^30^ utilized this method to simulate the dynamic crack propagation process under the initial stress state. The results demonstrated that the LS-DYNA element deletion method can well describe the formation of fractured regions and the propagation of radial cracks.

In this study, numerical simulation was conducted to analyze the development law of fractures in composite strata of soft and hard rock interlaced under the combined action of explosion stress wave and explosion gas. First, the propagation law of stress waves at the composite interface was studied, and the projected and reflected stress coefficients of stress waves were provided. Additionally, a two-dimensional single-hole model and a three-dimensional deep-hole blasting model were established. The propagation law and fracture distribution of stress waves in composite rock layers were explored. Moreover, the rationality of the projection and reflection coefficients was verified.

## Propagation law of stress waves in non-uniform continuum

**Figure 1:**
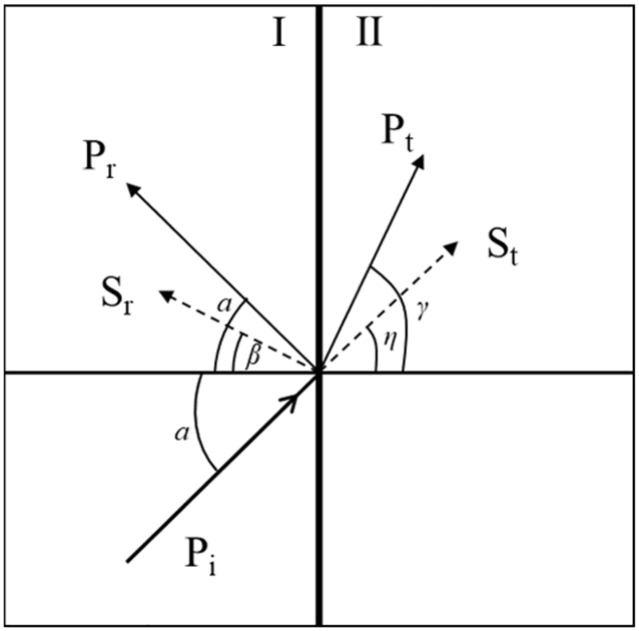
P_i_ incident in inhomogeneous medium.

when an elastic wave interacts with two elastic half-space interfaces, the incident wave P_i_ will be reflected and refracted at the discontinuity of the medium after incident on the interface, resulting in four new waves (Fig. 1): reflected longitudinal wave P_r_, reflected transverse wave S_r_, refracted longitudinal wave P_t_ and refracted transverse wave S_t_. The shear waves S_r_ and S_t_ are generated by the interaction of the incident wave P_i_ with the reflected wave P_r_, this phenomenon is called mode conversion of waves.

According to Snell's law, the wave speed is related to the angle of incidence, reflection and refraction as follows:

1 $$ \frac{{c_{P}^{{\text{I}}} }}{sin\alpha } = \frac{{c_{S}^{{\text{I}}} }}{sin\beta } = \frac{{c_{P}^{{{\text{II}}}} }}{sin\gamma } = \frac{{c_{S}^{{{\text{II}}}} }}{sin\eta } $$

**Figure 2:**
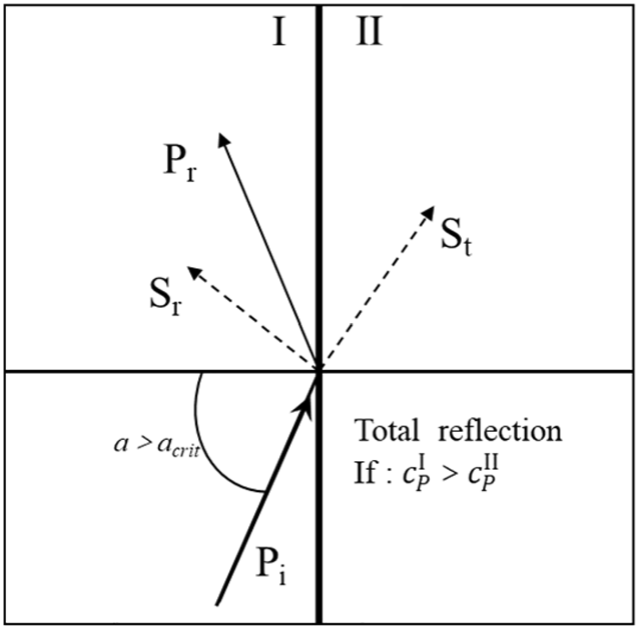
Total reflection of P_i_.

For the incident P wave, when the velocity of the P wave in the projected rock mass is greater than the velocity of the P-wave in the incident rock mass, there is a critical angle, and the critical angle α_*crit*_ can be calculated by Eq. (2). When α > α_*crit*_, total reflection occurs and the refracted longitudinal wave P_t_ disappears in medium II. Figure 2 illustrates the propagation of incident P-waves interacting with the rock interface at incident angles greater than the critical angle.

2 $$ \alpha_{crit} = arctsin\left( {c_{P} /c_{p}^{*} } \right) $$

**Figure 3:**
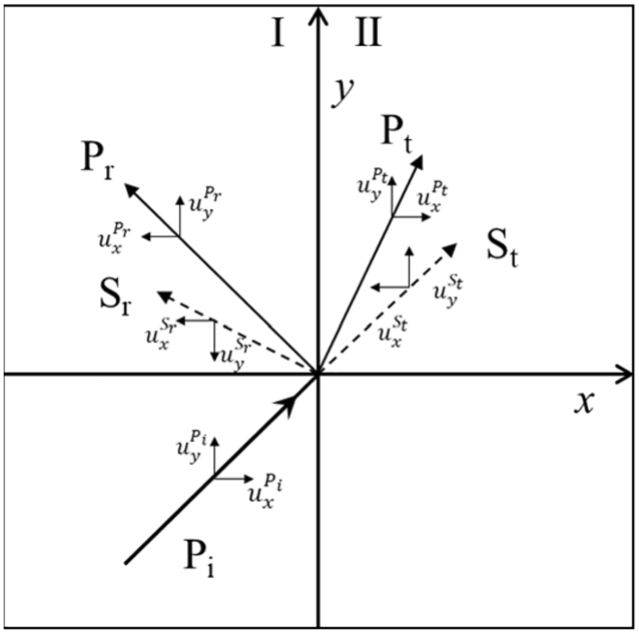
Displacement component.

It is assumed that the rock interface is continuous, that is, the interface does not move relatively when the stress wave passes through, and the displacement and stress generated by the explosion stress wave at the interface are continuous. Figure 3 shows the displacement component after the longitudinal wave P_i_ is incident on the continuous interface at angle α, and the boundary conditions of displacement continuity (i and ii) and stress continuity (iii and iv) are satisfied:

3 $$\begin{aligned} {\text{(i)}}\quad u_{y} &= u_{y}^{*} \Rightarrow u_{y}^{{P_{i} }} + u_{y}^{{P_{r} }} + u_{y}^{{S_{r} }} = u_{y}^{{P_{t} }} + u_{y}^{{S_{t} }}\\ {\text{(ii)}}\quad u_{x} &= u_{x}^{*} \Rightarrow u_{x}^{{P_{i} }} + u_{x}^{{P_{r} }} + u_{x}^{{S_{r} }} = u_{x}^{{P_{t} }} + u_{x}^{{S_{t} }}\\ {\text{(iii)}}\quad \sigma_{x} &= \sigma_{x}^{*} \Rightarrow \sigma_{x}^{{P_{i} }} + \sigma_{x}^{{P_{r} }} + \sigma_{x}^{{S_{r} }} = \sigma_{x}^{{P_{t} }} + \sigma_{x}^{{S_{t} }}\\ {\text{(iv)}}\quad \tau_{x} &= \tau_{xy}^{*} \Rightarrow \tau_{xy}^{{P_{i} }} + \tau_{xy}^{{P_{r} }} + \tau_{xy}^{{S_{r} }} = \tau_{xy}^{{P_{t} }} + \tau_{xy}^{{S_{t} }} \end{aligned} $$

The reflection and transmission coefficients for the calculated stress can be determined from the above boundary conditions:

4 $$ \begin{array}{*{20}c} {{\text{P}}_{{\text{r}}} } & {\quad {\text{S}}_{{\text{r}}} } & {\quad {\text{P}}_{{\text{t}}} } & {\quad {\text{S}}_{{\text{t}}} } \\ {R_{{P_{r} }}^{\sigma } = \frac{{\sigma^{{P_{r} }} }}{{\sigma^{{P_{i} }} }}} & {\quad R_{{S_{r} }}^{\sigma } = \frac{{\tau^{{S_{r} }} }}{{\sigma^{{P_{i} }} }}} & {\quad T_{{P_{t} }}^{\sigma } = \frac{{\sigma^{{P_{t} }} }}{{\sigma^{{P_{i} }} }}} & {\quad T_{{S_{t} }}^{\sigma } = \frac{{\tau^{{S_{t} }} }}{{\sigma^{{P_{i} }} }}} \\ \end{array} $$

**Figure 4:**
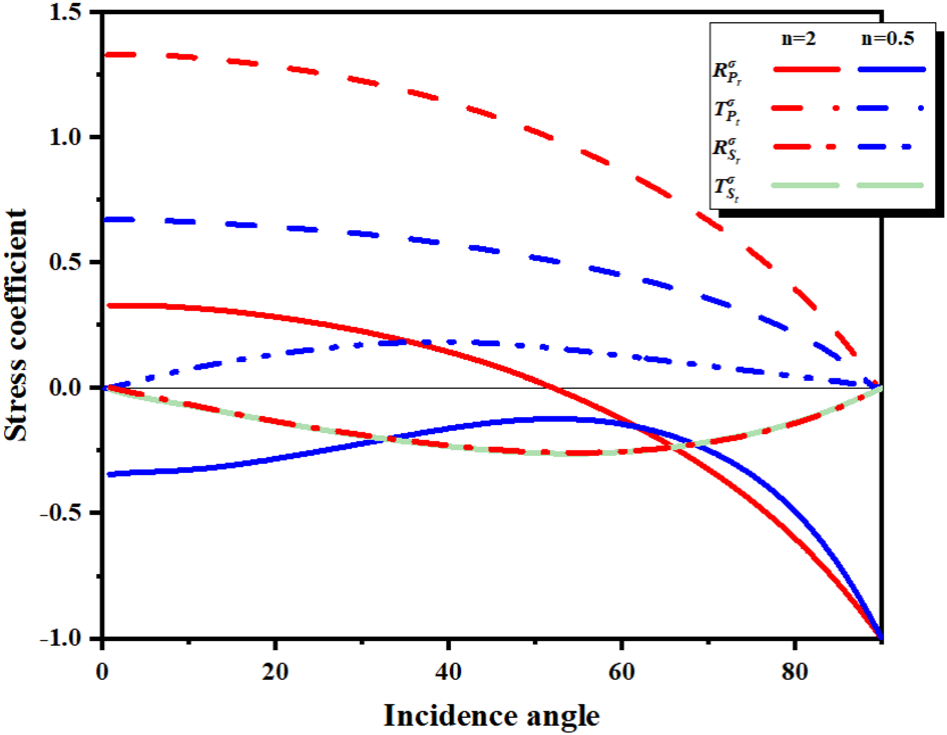
Stress coefficient at different incident angles.

Figure 4 shows the stress reflection coefficients of the two wave impedances at different incident angles. The refracted wave intensity depends on the acoustic impedance ratio n of the incident medium and the projection medium, and the wave impedance is determined by Eq. (5).

5 $$ n = \frac{{\rho^{II} c_{P}^{II} }}{{\rho^{I} c_{P}^{I} }} $$

For explosive compressional waves incident on rock materials, a positive reflection coefficient produces compressional waves, and a negative reflection coefficient produces tensile waves. When the incident compression wave is incident from the hard rock to the soft rock, n < 1, $R_{{S_{r} }}^{\sigma }$, $T_{{S_{r} }}^{\sigma }$ > 0, $T_{{P_{t} }}^{\sigma }$ $R_{{P_{r} }}^{\sigma }$ < 0; when the incident compression wave is incident from the soft rock to the hard rock, n > 0, $R_{{P_{r} }}^{\sigma }$ > 0, $T_{{P_{t} }}^{\sigma }$, $T_{{S_{r} }}^{\sigma }$ < 0, $R_{{P_{r} }}^{\sigma }$ increases with the increase of the incident angle, and the reflected compressional wave is converted into a tensile wave.

## Research methods and model parameters

### Model and material

**Figure 5:**
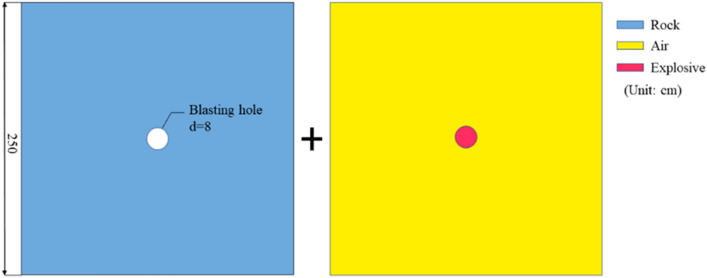
Numerical model of single blasting.

In order to verify the accuracy of the model, a single-hole blasting simulation is firstly carried out. Figure 5 shows the model for simulating the blasting process of a single hole. The thickness of the model is 1 cm. The fixed strain parameter is set in the normal direction of the model plane to simulate the quasi two-dimensional plane problem; the non-reflection boundary is set around the model to simulate the infinite rock medium. Taking the ideal gas model as the fluid. The fluid–structure coupling algorithm was used to simulate the crack propagation law of rock under the coupling action of blasting stress wave and gas during single-hole blasting.

The HJC model was been adopted in this paper for rock constitutive model, which considering the effect of strain rate and damage accumulation and can well describe the crushing behavior of elastic–plastic materials under dynamic loads. In addition, the LS-DYNA commercial software provides the keyword *MAT_ADD_EROSION to add failure criteria to the rock model, which can simulate the crack propagation process during the explosion by deleting cells. The parameters of the maximum tensile stress *p*_max_ and the maximum shear strain γ_max_ are chosen to well describe the growth and evolution of explosion-induced cracks^27^. The parameters of the rock HJC constitutive model are given in Table 1. The parameters of the hard rock model are quoted in the literature^31^, and the soft rock model is quoted in the literature^32^.

**Table 1:** Constitutive model parameters of soft and hard rock.

Parameter	Valye		Parameter	Valye		Parameter	Valye	
	Hard	Soft		Hard	Soft		Hard	Soft
$\rho_{0}$ (kg/m^3^)	2600	2416	*S*_*max*_	5	7	*K*_*1*_	12.87	8.1
*G* (GPa)	28.70	5.16	*T* (MPa)	12.2	8.7	*K*_*2*_	16.31	9.1
*f*_*c*_ (MPa)	119	88	*D*_*1*_	0.04	0.013	*K*_*3*_	64.95	89
*N*	0.86	0.79	*D*_*2*_	1	1	*FS*	0.035	0.02
*A*	0.28	0.32	*P*_*crush*_ (MPa)	41	29	*μ*_*crush*_	1.24E−3	16.2E−3
*B*	2.5	1.76	*P*_*lock*_ (GPa)	1.2	0.8	*μ*_*lock*_	0.011	0.012
*C*	0.0018	0.0127	*EF*_*min*_	0.01	0.0046			

The explosives model is modeled using the JWL equation of state, and the parameters of the explosives model are calibrated by Tawadrous^33^.

6 $$ P_{J} = A_{J} \left( {1 - \frac{\omega }{{R_{1} V}}} \right)e^{{ - R_{1} V}} + B_{J} \left( {1 - \frac{\omega }{{R_{1} V}}} \right)e^{{ - R_{1} V}} + \omega E_{0} /V $$

where P_J_ is the detonation wave pressure, V is the relative volume of the detonating gas, E_0_ is the specific internal energy, A_J_, B_J_, R_1_, R_2_ and are the JWL model parameters, and air is described by the ideal gas law. The plugging material in the blasting-hole is modeled by a soil material model (*MAT_SOIL_AND_FOAM) whose parameters are determined by Wang et al.^34^.

### Blasting process of single-hole model

The single-hole blasting process of rock can be divided into three stages.

**Figure 6:**
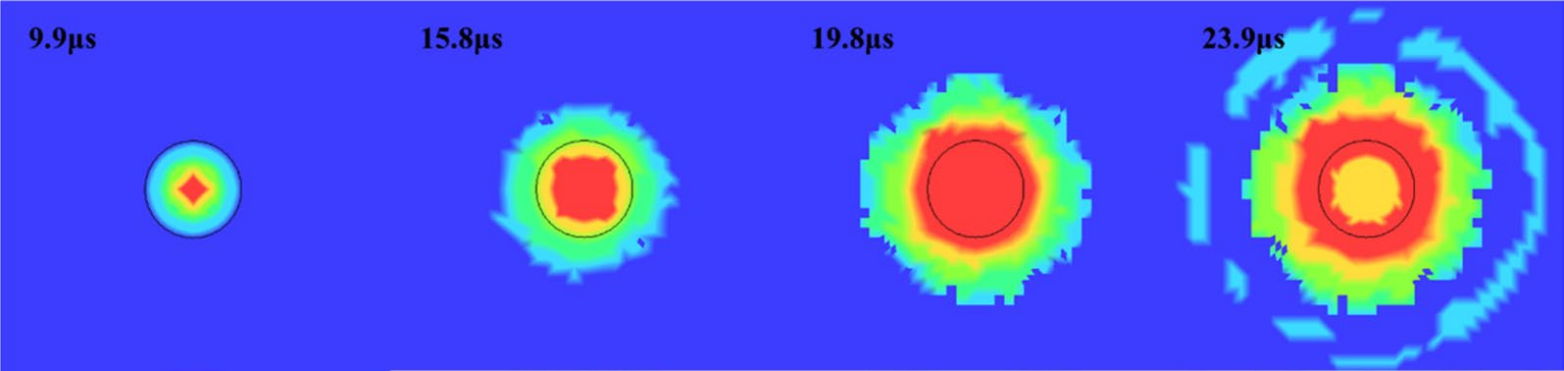
Explosive detonation process.

Stage 1 is the detonation wave action stage, as shown in Fig. 6. This stage lasts for 23.8 μs. The detonation wave generated by the detonation of the explosive acts on the inner wall of the blasting-hole and smashes the surrounding rocks. At this time, the propagation speed of the detonation wave is higher than the propagation speed of the compressive stress wave in the rock and basically consistent with the expansion speed of the rock failure circle. After the detonation process is over, the surrounding rock generates the first stress wave and propagates around away.

Stage 2 is the high-pressure gas action stage, the high-pressure gas generated by the explosion of the explosive continues to act on the surrounding rocks, which makes the scope of the crushing circle continue to expand. When the pressure is reduced to the dynamic compressive strength of the rock, the diffusion of the crushing zone stops, leaving a circular cavity.

**Figure 7:**
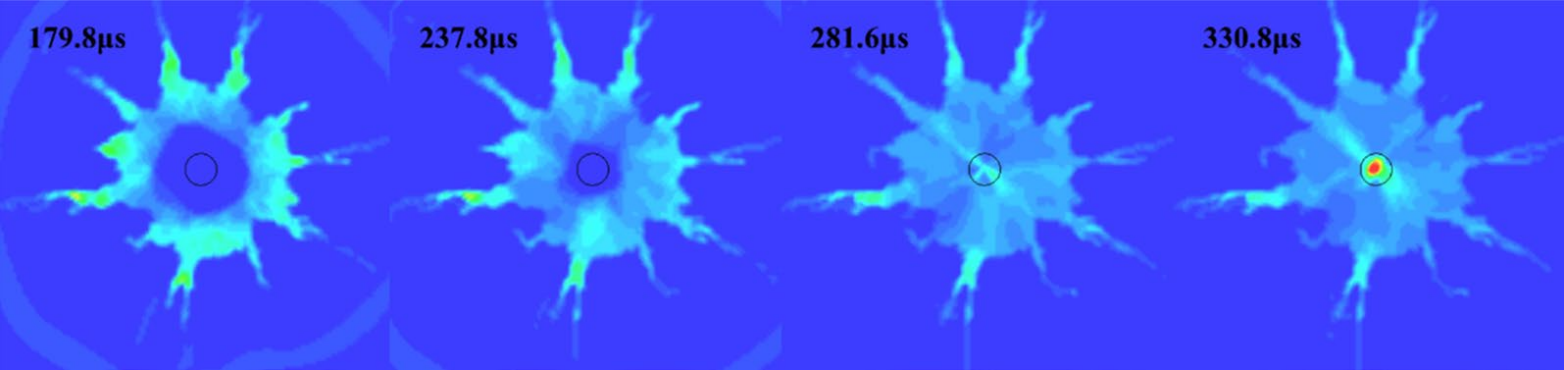
Explosive gas reflected in the blast hole.

**Figure 8:**
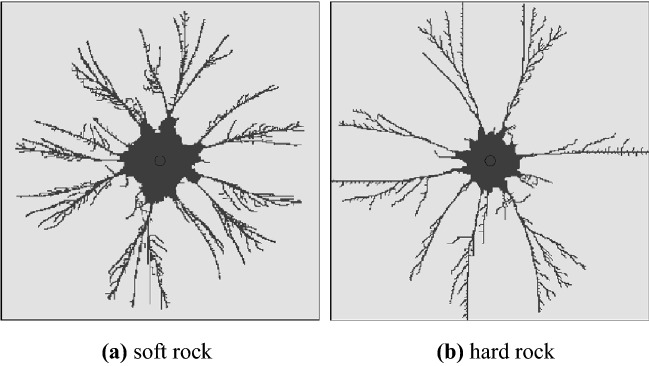
Simulation results of burst cracks.

**Figure 9:**
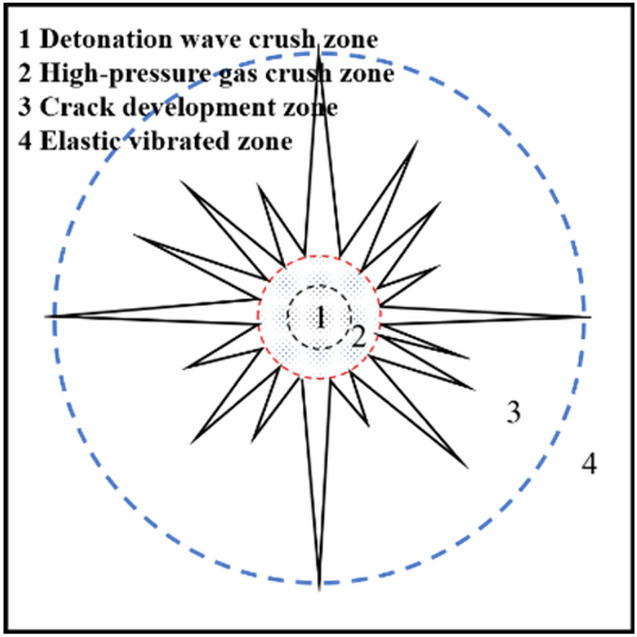
Theoretical results of single-hole blasting.

Stage 3 is the explosive gas action stage, the high-pressure gas generated after the explosion is continuously reflected in the crushing circle (Fig. 7) and continues to exert pressure on the surrounding rock mass, and the tensile cracks continue to expand under the action of the gas wedge. When the energy in the air is not enough to induce new cracks, the blasting process ends, leaving an obvious broken circle and crack development circle around the blast hole. Figure 8 shows the simulated fracture results of hard rock and soft rock. The development of fractures in soft rock is significantly larger than that in hard rock. The fracture circle range in the model is about 3–5 times the diameter of the blasting-hole, which is similar to the theoretical result (Fig. 9).

### Stress propagation in the single-hole blasting model

The empirical formula for the attenuation law of shock wave and stress wave pressure in rock is:

7 $$ P_{R} = P_{0} \left( {\frac{r}{{r_{b} }}} \right)^{ - \alpha } $$

where *P*_*R*_ is the shock stress wave pressure, *R* is the distance from the blast hole, *r*_*b*_ is the blast hole radius, and α is the pressure decay index. *α* = 2 + *μ*_*d*_/(1 − *μ*_*d*_) for shock waves and *α* = 2 − *μ*_*d*_ (1 − *μ*_*d*_) for stress waves, where *μ*_*d*_ is the dynamic Poisson’s ratio of the rock.

Under the condition of the cylindrical coupling charge, the initial pressure *P*_*0*_ of the shock wave into the rock is^35^:

8 $$ P_{0} = \frac{{2\rho_{e} C_{d}^{2} \rho_{0} C_{p} }}{{(1 + \gamma )\;\;(\rho_{0} C_{p} + \rho_{e} C_{d} )}} $$

*ρ*_e_, *C*_*p*_, *C*_*d*_ and *γ* are the density of the explosive, the sound velocity of the rock, the detonation velocity of the explosive and the adiabatic expansion coefficient of the detonation product, respectively, and *γ* is generally taken as 3.

**Figure 10:**
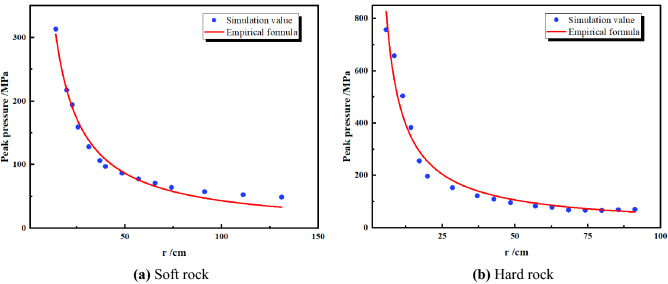
Peak pressure decay.

In the single-hole model, take the measuring cell along the direction of the blast hole and record the peak pressure. As shown in Fig. 10, the decay trend of the simulated values is basically consistent with the empirical formula. The stress decay, stress wave and crack development of the model are combined with realistic laws, and reliable conclusions can be drawn from the numerical simulation.

## Numerical simulation of explosion stress wave and fracture development in composite rock formation

### Propagation law of blast stress wave in composite rock interface

**Figure 11:**
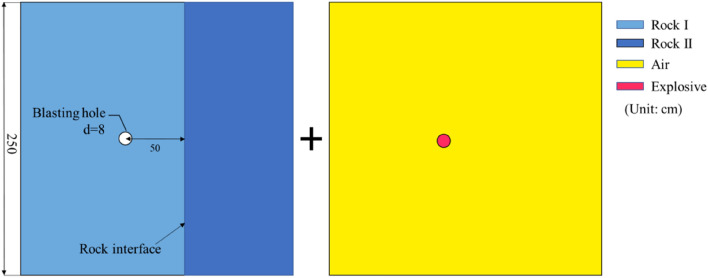
2D model of single-hole blasting in composite rock formation.

The two-dimensional model of single-hole blasting in composite rock formation show in Fig. 11. The distances between the center of the blast hole and the interface are set to 25 cm, 50 cm, and 75 cm, respectively. In the model, the contact control of automatic surface contact is set at the interface between rock layer I and rock layer II. The thickness of the contact surface is ignored, and the interface stiffness is determined by the elastic modulus of the material and the element size. The static coefficient of friction between the rocks is 0.08 and the dynamic coefficient of friction is 0.2. The other model parameters are the same as the single-hole blasting model.

**Figure 12:**
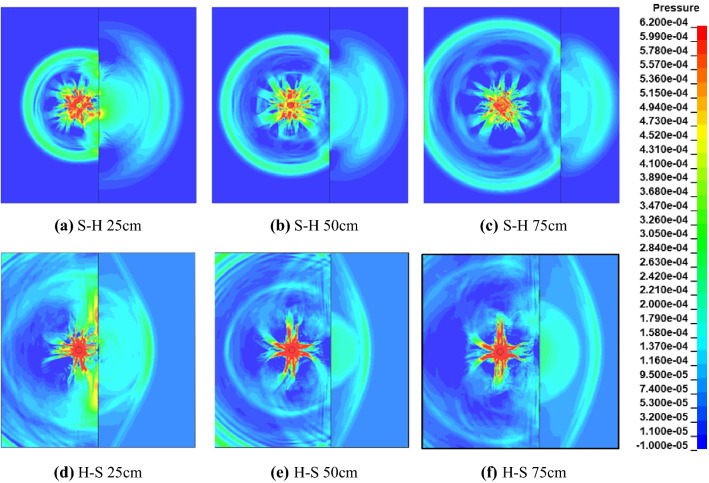
Stress cloud diagram of single-hole blasting model of composite rock formation.

For two-dimensional waves in the plane, Fig. 12 shows the stress cloud diagram of the single-hole blasting model of the composite rock layer. S–H refers to the incident wave entering the hard rock from the soft rock, and 25 cm refers to the distance between the blast hole and the rock interface. The blast stress wave is refracted and reflected at the rock interface, and produce reflected and refracted waves in soft rock and hard rock respectively. Similar to the theoretical study, compressional reflection waves are generated in the S–H model, while tensile reflection waves are formed in the H–S model. Due to the different propagation speed of stress wave in hard rock and soft rock, the waveform of refracted stress wave changes, showing that the wavelength is elongated in soft rock and shortened in hard rock.

In Fig. 12a–c, when the incident angle of the incident wave in the soft rock exceeds the critical incident angle, total reflection will occur at the interface. At this time, the incident wave will no longer generate a refracted wave at the interface. As the refracted stress wave continues to propagate, the wave-front in the hard rock is separated from the incident wave-front in the soft rock. In Fig. 12d–f, the H–S model, there is no critical reflection angle when the stress wave propagates from the hard rock to the soft rock, and the refracted wave and the interface S_t_ wave will overlap at the interface. In addition, it can be seen from Fig. 4 that the refracted stress wave will continue to decrease and the reflected stress wave will continue to increase as the incident angle increases, which is reflected in both the S–H and H–S models. Subsequent pressure waves from high-pressure gas will create a region of persistent compressive stress at the rock interface, the size of which is related to the waveform at the interface.

**Figure 13:**
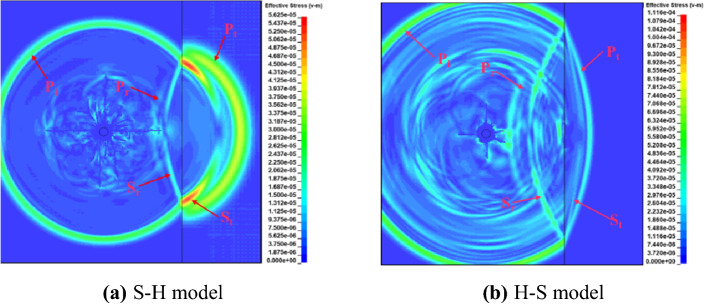
Effective stress cloud diagram of blasting model.

In order to better observe the propagation law of the blast stress wave at the rock interface, the air part in the model is removed to eliminate the influence of the stress wave formed by the reflection of high-pressure air. Figure 13 shows the effective stress nephogram of the model, when the distance between the center of the blast hole and the interface of hard and soft rock is 75 cm.

Since the propagation velocities of S_t_ and S_r_ waves are different from those of P_t_ and P_r_ waves in the soft rock incident hard rock model, the surface waves S_t_ and S_r_ generated by the incident wave and the refracted wave at the rock interface can be clearly observed in Fig. 13. Contrary to P_t_ and P_r_, the intensity of the surface wave increases or decreases with the increase of the incident angle, showing that the surface wave generated farther from the blasting-hole is stronger. In the soft rock incident hard rock model, the Sr wave generated by the refracted P_t_ wave is preferentially propagated by the blast stress wave in the hard rock, and the S_t_ wave and the refracted stress wave are separated at the interface. This phenomenon is consistent with the experimental results of Rossmanith and Fourney^36^. In the model where the hard rock is incident on the soft rock, the four new waves generated by the incident wave coincide at the interface.

**Figure 14:**
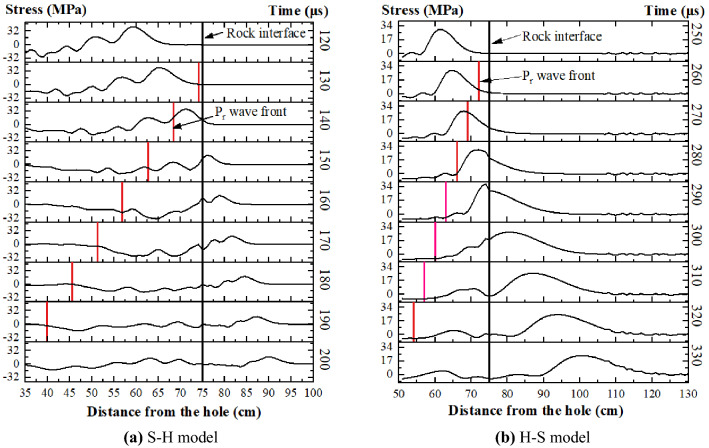
Explosion Stress waveform.

**Figure 15:**
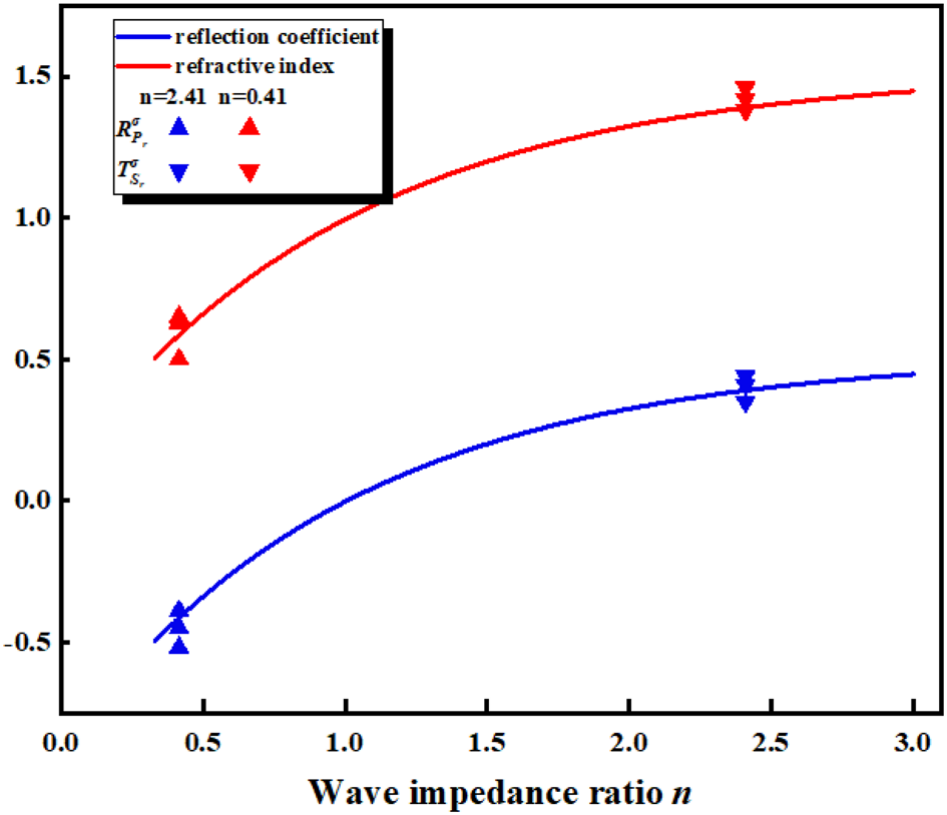
Reflection and refraction stress coefficients when the incident angle is 90°.

When the compression wave P_i_ is vertically incident on the rock interface, the stress coefficient $T_{{S_{r} }}^{\sigma }$, $T_{{P_{t} }}^{\sigma }$ is 0, and only refraction and reflection occur. Extract the stress value of the cell perpendicular to the center of the interface in the model and draw the stress waveform diagram (Fig. 14). At this time, after the incident wave propagates to the interface, the refracted wave is on the right side of the interface, and the incident wave and reflected wave are superimposed on the left side of the interface. according to the initial incident stress wave and the stress wave attenuation formula (Eq. 8), the reflected wave waveform can be separated, and the stress value of the stress wave peak and the stress coefficient can be obtained. The wave velocity in hard rock is 5780 m/s, and the wave velocity in soft rock is 2586 m/s. The acoustic impedance ratios n^H–S^ and n^S–H^ are 2.41 and 0.42, respectively. Figure 15 shows that the refraction and reflection coefficients in the model are basically consistent with the theoretical curves.

### Explosion crack propagation law of composite rock strata interface

**Figure 16:**
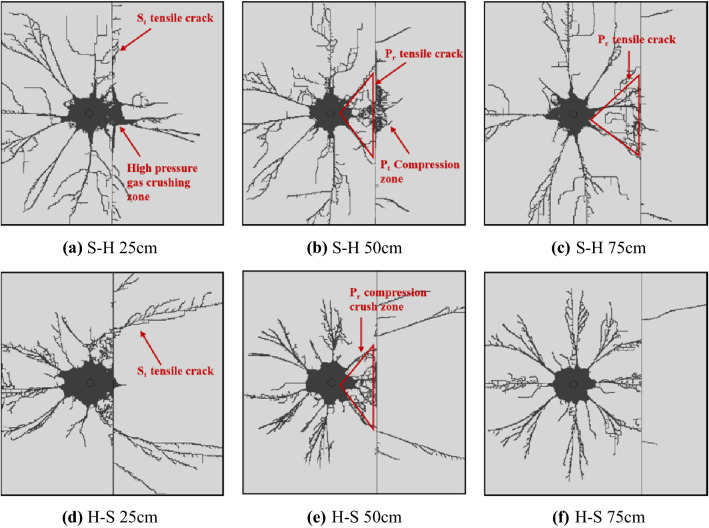
Crack propagation under different incidence modes of P_i_.

The final propagated cracks for the H–S and S–H models show in Fig. 16. The distances from the center of the blasting-hole to the rock boundary are 25 cm, 50 cm and 75 cm, respectively. In models H–S 25 and S–H 25, since the explosion point is close to the interface, the rock at the other end of the interface has a crush zone created by the high pressure gas. In the S–H 50 and S–H 75 models, at the interface near the blast hole, a triangular fragmentation area formed by reflected compression waves appears in hard rocks, and a crush area formed by projected compression waves appears in soft rocks. The ends form tensile cracks formed by surface P_r_ compression waves. In the H–S 50 and H–S 70 models, due to the interaction of the reflected compressive stress wave and the incident compressive stress wave, the soft rock at the interface appears a crushed area that develops parallel to the interface, thus forming a new reflection interface to cause a fractured area. Tensile cracks also appear in this model. The crack position is related to the critical angle. When the incident wave exceeds the critical angle, the refracted compressional wave is separated from the interface tensile wave. The tensile stress formed by the interface breaking S_t_ reaches a peak value and is accompanied by tension The appearance of elongation cracks.

### Propagation and crack propagation of blast stress wave in bench deep hole blasting in composite rock

**Figure 17:**
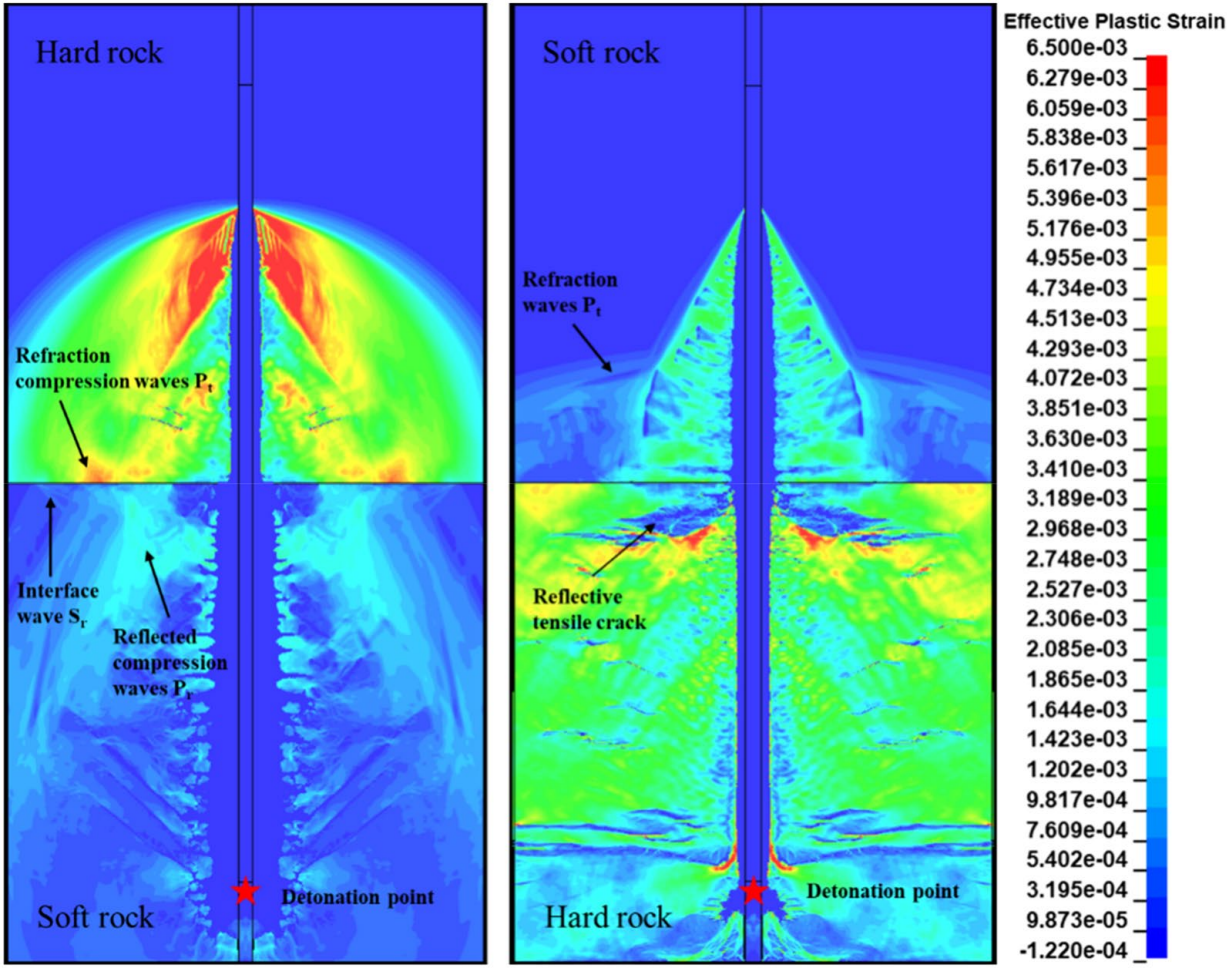
Stress cloud diagram of 2D model of deep hole blasting.

The simulation results of the two-dimensional model of step blasting shows in Fig. 17. The detonation wave generated by the detonation of the columnar explosive forms a conical compression wave front in the rock mass. As the wave front propagates, the stress wave intensity will attenuate, and the propagation velocity will also decay. Due to the propagation speed of the stress wave in the rock medium, the cone angle of the wave front is also different, and the angle out of the soft rock is smaller than that of the hard rock. In addition, due to the reflection of high-pressure gas in the blast hole, a stretched area will be generated behind the wave front. The model shows radial cracks scattered away from the initiation point near the initiation point, and the crack angle in soft rock is larger than that in hard rock. These phenomena are consistent with the research conclusions of Uenishi and Rossmanith^37,38^.

When the wave-front is incident in the soft rock, the crushing zone generated in the soft rock by the detonation wave-front reflected compression wave and the incident wave can be observed in the model. When the explosive is detonated in hard rock, tensile cracks generated by reflected compressional waves appear in the hard rock at the rock interface, and transmission waves that propagate in preference to the blast stress wave also appear in soft rocks.

**Figure 18:**
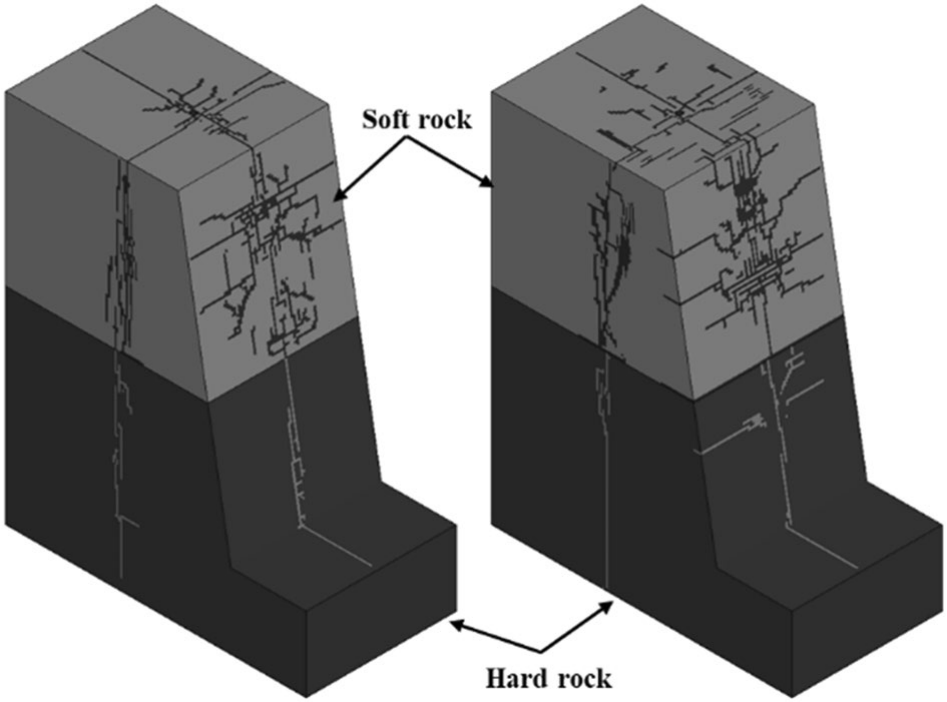
Development of step explosion crack.

**Figure 19:**
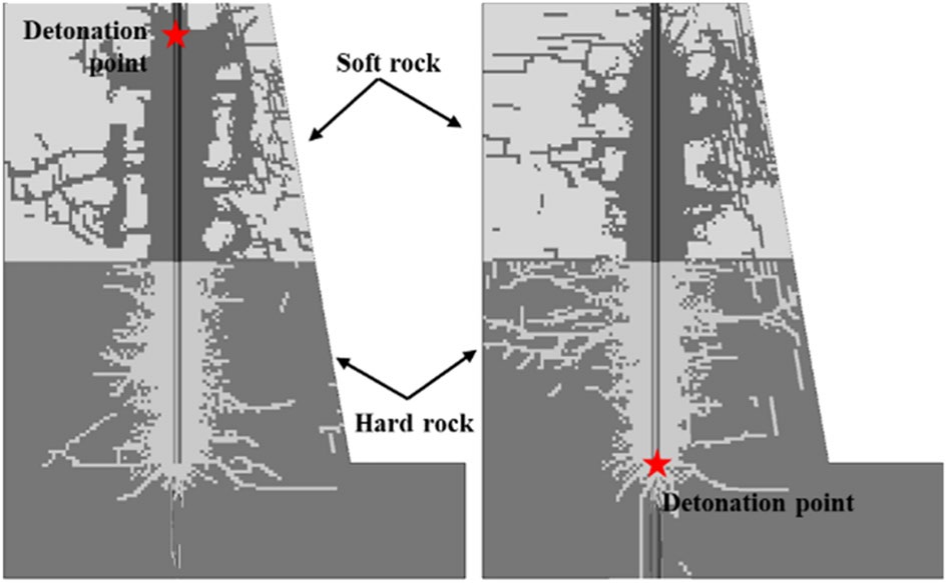
Step section.

In order to study the blasting effect of the initiation position on the bench in the composite rock, a three-dimensional bench deep hole model was established. Figures 18 and 19 show the crack development of the 3D blasting model.

Similar to the two-dimensional model of single-hole blasting in composite rock, when the conical compression wave is incident from the soft rock to the rock-stratum interface, a crushing zone will be generated in the soft rock, and this zone will change the blast stress wave in the hard rock. It affects the development of cracks in the hard rock at the interface, resulting in the phenomenon of under-explosion in the step blasting. However, when the conical compression wave is incident from the hard rock to the interface of the rock layers, the reflected tensile wave will be generated, resulting in more tensile cracks in the hard rock and better fragmentation effect on the hard rock.

## Conclusions

The propagation and crack development laws of blast stress waves in soft-hard layered rock layers were explored using the HJC model. First, the refraction and reflection laws of incident stress waves at the continuous interface were analyzed. Then, the HJC model was introduced, and the single-hole blasting process was analyzed and verified. Through numerical simulation, the variation law of blast stress wave at the rock interface under the two incident modes of H–S and S–H was discussed, as well as the development law of cracks at different distances from the center of the blasting hole to the interface. Additionally, the action process of the detonation wave and the soft-hard interface in deep hole blasting and the development of cracks at the interface were investigated. The following conclusions can be drawn.

1. Under the condition of the interface between continuous soft and hard rock layers, the incident stress wave P_i_ generates four new waves during the interaction with the interface: the reflected wave P_r_, the refracted wave P_t_, the interface wave S_r_, and the interface wave S_t_. The stress changes generated by the four waves in the rock mass are associated with the incident mode and incident angle of the incident wave P_i_. In this study, this process of reproduced in LS-DYNA, and the performance of the calculation method of the stress coefficient is verified.
2. Both H–S and S–H incidents have a triangular fracture zone at the rock interface near the blast hole, while the generation mechanism is different. It is induced by a reflected tensile wave in the H–S model and a reflected compressional wave from a compressional wave at the interface in the S–H model. Tensile cracks caused by the surface wave St are generated at the interface of rock II in a direction away from the blasting hole, and the cracks decrease with the increasing distance from the blasting hole.
3. The detonation method of the continuous charge in deep hole blasting affects the propagation and crack development of the blast stress wavefront in the rock mass. Compared with a detonation in soft rock, a detonation in hard rock enables the blast wave front interaction with the interface to weaken the under-breaking phenomenon in the hard rock and improve the quality of the blasting operation.

## Data Availability

The experimental data used to support the findings of this study are included within the article.
